# Professional Ethics

**Published:** 1893-09

**Authors:** F. A. Roy


### PROFESSIONAL ETHICS.

TO THE EDITOR:

SIR,—After reading, in the *Dental Cosmos* for July, Dr. Dean's "Professional Ethics," and the editorial called forth by it, one asks, How shall we get that dental code which shall be ethical in that it subserves the welfare of the community, including the dentists? One sees two things showing the present condition of formulated dental ethics: 1, a more or less generally adopted code of ethics, but with a growing sentiment for changing or even dropping it; 2, a certain amount of legislation differing in various localities. By all means we should have our code of ethics emanating from the most representative associations as the expression of our best men. There we will write ourselves down at what we are, and the community can and will judge us by it. But the community has no voice in formulating this code which represents our correct relation to that community. And how can it be enforced? We may punish from a professional stand-point by censure or expulsion from association, but this does not protect the community. Now, when it comes to legislation, the community has its voice, and powerful enough, as the editorial says, to cause the failure of such legislation as benefited the dentist primarily, and the community can enforce its laws if it will.

Therefore, would it not be best to let our code of ethics be a simple declaration of principles only which are comparatively easily formulated to suit all localities and put out greater exertions towards getting the laws of the community so made that they can be appealed to to decide matters just as in any line of life, business or professional? In this way the community is protected and can have its voice, and as legislation may be enforced, laws can be tried positively and the resultant experience of their practical operation will decide further changes or additions, and in the end our code of ethical principles may even change in the light of that experience. The adoption of a code has not rid us of unprofessional acts. The completion or extension of legislation will not; but it will give something tangible to control or at least punish wrong conduct, thus acting for the welfare of the community, including the dentist.

Yours fraternally,

F. A. Roy.

